# Medical and Philosophical Intelligence

**Published:** 1816-11

**Authors:** 


### MEDICAL AND PHILOSOPHICAL INTELLIGENCE.

THE College of Physicians, in order to be more exact in giving general directions for dispensing medicines, endeavoured to do away the practice of dropping fluids from bottles, by substituting the graduated minim measure; but forgot to acquaint us of the very great difference existing between the minim and drop, which we were led to think, and indeed it was generally thought to be synonimous. But from this mistake the greatest errors are likely to accrue. Many practitioners invariably order the minim, but in the same number as they had formerly directed to be dropped from the lip of a bottle, and by this I am led to believe many patients have taken twenty drops of a medicine, when ten only was the intended dose, which, in the exhibition of many medicines, would be of the most serious and alarming consequences.

The following Table is intended to shew how greatly we may err in the general use of the minim measure.

*** These fluids were all dropped from a two-ounce bottle, containing f. ℥j. from which I was enabled to drop exactly. The lip of the bottle was 3/[illegible] inch wide.

| | | | |
|---|---|---|---|
| Aqua Pura gtt. xij. | Took up the space of | ♏ x | In the graduated minim measure, by Mr. Lane, procured from Apothecaries' Hall. |
| Tinct. Opii gtt. xxvj. | | ♏ x | |
| Acid Sulp. D. gtt. xxx. | | ♏ xv | |
| Tinct. Kino gtt. xxxv. | | ♏ xxv | |
| Æther Rectif. gtt. xxx. | | ♏ xv | |
| Vin. Antim. gtt. xxx. | | ♏ xx | |
| Liq. Arsenic gtt. xx. | | ♏ x | |
| Ol. Lavend. Ess. gtt. xx. | | ♏ x | |
| Acid Mur. gtt. xv. | | ♏ xx | |
| Vin. Opii gtt. xxxij. | | ♏ xxv | |
| Liq. Potassæ gtt. xxiv. | | ♏ xv | |

Hence, if a physician wishes to give his patient a dose of Liq. Arsenical. (of ten drops), and orders ♏ x. it is measured by this graduated minim measure, and, instead of ten drops, he takes twenty; and it is impossible, from this, to suppose that the medicine will have its desired effect, or perhaps it may be continued so long as to cause death!! Who is then to be blamed?

It must be hoped, that, from the preceding observations, which must appear obvious to all, that the minim will not be used, as, although it may be an addition, it can be no way counted an improvement, in the utensils of an apothecary's shop.

(Signed) HENRY WARD.

*Letter from* Mr. GRAINGER, *giving an Account of a Ball found in the Heart of a Buck.*—On August the 31st past, a buck that was remarkably fat and healthy in condition, was killed in Bradby Park,

Park, and, on opening him, it was discovered that, at some distant time ago, he had been shot in the heart; for a ball was contained in a cyst in the substance of that viscus, about two inches from the apex. The surface of the cyst had a whitish appearance. The ball weighs 292 grains, and was beat quite flat. What led to the discovery of the ball was the unusual circumstance of a firm adhesion of the heart with the pericardium to the ribs of the left side. One of the ribs at the place of the adhesion, bore marks of having been fractured. Mr. Richardson, the park-keeper, who opened the animal, and communicated the account to me, is of opinion that the ball had struck some hard substance before entering the body of the deer, as it could not otherwise have become so flat. In the second volume of the Medico-Chirurgical Transactions, is published an extraordinary case of a soldier who survived forty-nine hours after receiving a bayonet-wound of the heart; but the above particulars of a gunshot-wound of the heart will afford a still more striking example of the great extent to which this vital organ may sustain an injury from external violence, without its functions being immediately destroyed, or even permanently impaired. The recovery from an accident of this nature having happened to an animal of an inferior order to that of man, can in no respect materially vary the physiological import of the fact; therefore this authentic statement of it may be worthy of a place in that department of your Journal allotted to compendious articles of intelligence. *Burton-upon-Trent, September 7, 1816.—Edinb. Med. and Surg. Journ.*

---

*Edinburgh Dissection of the Brain.*—A considerable interest having been excited in Edinburgh, for some time past, about the discoveries of Gall and Spurzheim, relative to the structure and functions of the brain, Dr. Spurzheim at length made a dissection of that organ, in one of the anatomical dissecting rooms of the University. Besides the regular students, many of the professors were present, as well as other scientific persons interested in these new and important discoveries. Dr. S. succeeded in making the most perfect dissection of the brain, and received the approbation of those who were present. Many persons who had previously opposed the new method of dissecting this organ, testified to the superiority of Dr. Spurzheim's new mode of developing the hitherto unexplored structure of the brain and nervous system.—*Phil. Mag.*

---

At a meeting of the Common Council, held at the Council House in the city of Bristol, on Wednesday, the 10th day of July, 1816,—on the motion of Sir William John Struth, Mayor of the said city, it was agreed and ordered unanimously, that the freedom of the city be presented to William Henry Goldwyer, Esquire, as a token of their approbation of the important services rendered by him as surgeon to the Hospital for the Cure of Diseases in the Eyes, established in that city; and that the city seal be affixed to the instrument of admission.

The following Notice to Medical Students has been circulated to the various hospitals and medical lecturers in the metropolis.

"*Apothecaries' Hall.*

"*September* 26, 1816.

"The Court of Examiners have determined that all those persons who are now about to prosecute their studies in London with a view to practise as Apothecaries in England or Wales shall, before they are admitted to examination, produce a certificate of attendance for *six months at least* on the Physician's Practice of some Public Hospital, Infirmary, or Dispensary; and that all those students who have obtained certificates of attendance on lectures and hospital practice *previously* to the date hereof, shall be admitted as heretofore. (By order of the Court),

"JOHN WATSON, Secretary."

---

One of the Journals speaks of an operation performed on sheep for *Hydrocephalus internus*. Before these operations are proposed we should have better information concerning the disease. Trepanning sheep for hydatids is a very common and very ancient practice, when the animals are what is termed giddy; in which case, the hydatid is either extracted or punctured. We pretend to say, that the ventricles of a sheep's brain may not be cut into or tapped, and we conceive it unnecessary to guard any of our readers against attempting such an operation in the human subject; but accurate information is always important, and most so in pathology, where the difficulties of gaining it are the greater.

---

*A Chemical Analysis of the Moira Saline Water*; by Mr. ACCUM, Chemist, London.

| A wine gallon of the water contains, | Grains. |
|---|---|
| Muriate of soda | 1838 |
| Muriate of Magnesia | 210 |
| Muriate of Lime | 172 |
| Carbonate of Iron | 18 |
| Sulphate of Lime | 96 |
| Sulphate of Soda | 130 |
| Carbonate of Lime | 42 |
| | 2506 |

---

(*From our Parisian Correspondent.*)

*On Legal and Judicial Chemistry*; by Dr. CHAUMETON.—Considered in themselves, the Sciences are entitled to our homage, their cultivation brings into action all the faculties of intelligence, and their discoveries attest the power of genius. They place man at an immeasurable distance above all other animals, and they even establish a marked difference, a gradation between individuals of the same species.

It has been repeated to satiety, that science has become popular,—nothing is more false; and I maintain, that all the truly learned men in the world united would form on the globe but a very small republic, republic, scarcely sufficient to people a city of the second order. We have then reason to cry with Solomon, *stultorum infinitus est numerus;* in fact, who can be more ignorant in general than a soldier, an artisan, a financier, or the lord of a small domain. Yet, of these classes are composed the great majority of the human race; and it is for such individuals, at once stupid and ungrateful, that all the treasures of the mind and imagination are developed. It was for them that Archimedes invented the pulley and the endless screws, and by them he was assassinated. It was for them that Socrates traced his divine code of logic and morals, and it was by them he was poisoned. It was for them Lavoisier created a new philosophy, and by them he was dragged to the scaffold. I could multiply instances, *ad infinitum,* of these deplorable examples of ingratitude, which, far from slackening the zeal and efforts of the philanthropist, seems to animate it, as we find persecutions inflame the courage of sectaries. The sage is happy when he makes a useful discovery, for, to the happiness of his fellow creatures, it is that he consecrates his learned studies.

Amongst the sciences, fertile in application, and from which society draws the most precious advantages, Chemistry indisputably holds the first rank; her lamp throws a new and vivid light on nearly all the arts; as Count Chaptal, in his admirable work of "Chemistry applied to the Arts," has clearly demonstrated.

Dr. Reines has not considered chemistry under the same point of view, consequently he has not pursued the same plan; his object was, to offer to the public a manual of legal and judicial chemistry, and he appears to have attained his end.

He commences by showing the necessity of such a work, and lays down with precision fundamental truths and general principles of chemical doctrines.

He traces a very interesting historical sketch, in which he proves that the legislature of all ages and all countries have occasionally had recourse to the philosophical sciences for the principal points of their codes. He afterwards determines the characters and points out the features of judicial chemistry and chemical police; he presents an enumeration of the objects to which this double chemistry extends its influence, of which I propose to offer the developement in this analysis.

The first and most important of functions is that of nutrition. If bread be not naturally a poison, it may become so by criminal adulterations; some mix plaster of Paris with the flour, and this crime is very common in Germany, where the same mills are employed to grind the corn and the gypsum for the farmers to fertilize their land; others use alum to render their bread whiter, and add jalap to neutralize the salt. The London bakers, it is said, sometimes use an oxide of bismuth; those of Paris prefer white lead, which is still more dangerous.

The accidents produced by bread made of spurred rye, have been described in a multitude of works, and M. Decandelle has, it is said, discovered discovered that the spurs is not any degeneracy of the vegetable, but a kind of mushroom.*

Butter is subjected to numerous sophistications, extremely varied: there is mixed with it tallow, oxides of lead, chalk, &c. and it is coloured with celandine; kept in copper vessels, it forms verdigrease, and contracts fatal qualities; though M. Remer asserts, that he has known butter become rancid, acquire a green colour, without containing a particle of copper.

If water has found some detractors, it has had also its panegyrists; it has been demonstrated that beneficent Nature had covered the greater part of the globe with this fluid, because it was of all substances the most necessary for animals in general, and man in particular. This incontestible pre-eminence would alone be sufficient to secure our homage; but, what will be our admiration, our enthusiasm, and our gratitude, if we observe, with Araneologist, Quatremere, and Disjonval, that we must seek in water the source, the origin of all languages, all the sciences, and all the arts? [and, according to an English pathologist, of all diseases? Edit.]

Dr. Remer, whose imagination is less exalted than that of M. Quatremere, considers water only as a beverage. The taste is, in this case, generally the best judge; a water agreeable to the palate is rarely injurious to the health; that of streams naturally salubrious may acquire deleterious and even mortal qualities. Certain artisans, as dyers for example, sometimes poison brooks and even rivers for a considerable distance by the solution of drugs employed as coloring matter. There is annually an epidemic dysentery at Helmstedt in the autumn, when the flax is steeped in the Aller.†

Of what materials ought water pipes to be composed?—Those of wood are the most economical and the easiest to repair, but they soon rot and communicate to the water a nauseous flavour. M. Marc fancies those of baked earth too fragile; yet, if thick and properly made, they will last for ages without injury. In pleading in favor of leaden pipes, Dr. Marc undoubtedly wished to tranquillize the inhabitants of Paris, where they are in general use; I am by no means of opinion with my estimable fellow labourer, I recollect, that the aqueducts of lead, blamed by Vitruvius, have frequently rendered water insalubrious, and sometimes mortal. In other respects, I perfectly agree with M. Marc, that cast-iron pipes are deservedly to be preferred to all others.

Milk, that delicious aliment, that nectar so wonderfully adapted to the weak and delicate organs of young animals, becomes a disgusting and murderous composition in the hands of an avaricious milkman. The milk sold at Paris is detestable; in the first place,

---

* Ergot, or spurred grain, is now generally considered innocuous. We have a further communication on this subject from America.—Edit.

† An Elementary Treatise on Legal and Judicial Chemistry; by William Germain George Remer, M.D. &c. &c.

watered watered by those who bring it, it is subjected to new sophistications by the fruiterers, whose frauds are as daring as they are numerous; not content with inundating it anew with water, they mix up with it meal, starch, bran-water, soap-water, &c.; but more infamous still are the Austrian milkwomen, who poison their milk (as it is said) with potash and lime, under the pretext of making it thicker and retarding the coagulation.

I have travelled much, and every where I have found the wine adulterated in a scandalous manner; in some countries they possess the art of making it without using a single grape. Composed of various ingredients by the industrious Hollander, it is, however, less pernicious than the wine sold by the publicans of Paris, who do not scruple to mix litharge with it. No matter to them how many victims they make, if they only escape the vigilance of the police.—Heaven forbid that science should become popular! I must confess, however, that ignorance has often led to fatal errors. It is related, that a young lady wishing to communicate a soft flavor to the wine her husband drank, poisoned it by too strong a dose of litharge, the danger of which she never suspected.*

The means by which adulteration may be detected in wine, brandy, beer, cyder, and vinegar, are enumerated by Dr. Remer, who gives the pre-eminence to the *proof liquor* of Hahnemann, which I shall transcribe, as it has been considered by M. Marc also as an almost infallible re-agent. Dissolve two drachms of tartaric acid in sixteen ounces of distilled water, at a medium temperature, after having added two ounces of stone brimstone reduced to a fine powder; the whole must be shaken in a large bottle for ten minutes without ceasing; the liquor must then be left at rest for half an hour, and pour it off clear into another bottle containing four ounces of tartaric acid in powder; when the liquor has stood twenty-four hours, it is to be poured off clear into small phials, and preserved for use.

The greater part of our culinary vessels are of copper, this metal undoubtedly possesses great advantages, it is very malleable and may be moulded into any form; it heats quickly, and preserves that heat long; but of all the useful metals it is the most dangerous; it oxidizes with a prodigious facility, and its oxide is a dreadful poison. The tinning with which copper vessels are generally covered, is liable to be attacked by various substances; it melts at a very low temperature, is deteriorated, detached, and is otherwise far from innocent in its nature. It is as M. Guersent elegantly observes, a light veil which conceals the danger, instead of being a true preservative, and it only inspires a security often fatal.

Iron vessels communicate to the food a black colour and disagreeable taste; those incontestibly the best, are made of pottery, the commonest are of a kind of argillaceous earth, mixed with

* A monk invented gunpowder; a bishop, bombs; a capuchin, lettres de cachet.—We owe to monks false titles, false charters;—all these blessings came from the same hands.

sand, sand, to render it more porous and more proper to bear the heat. It has been generally considered as absolutely necessary to glaze the inside of those vessels, but M. Remer has proved that this precaution is not indispensable; he has had made, under his own eye, unglazed vessels which resisted a violent degree of heat, and which did not absorb the water. It is true, that whatever is dressed in these vessels is more liable to be burnt, and they are besides liable to be penetrated by various fat substances. The pottery of Dr. Remer is, therefore, useful without being perfect,—to give it that perfection, it is requisite to find a material which unites the advantages without the inconveniences of black lead and copper, of which the common glazing is composed. We might, for example, (like the English) glaze them with sea-salt, but this method is impracticable in the greater part of our manufactures, where the fire is not sufficiently strong. M. Remer has pointed out the effects and the success of Niesemam, Mœller, Wagner, Darracq, Massieu, Westrumb, and Feilner, who have employed nitre, sea-salt, potash, fluor spar, pumice-stone, manganese, &c.

*(To be concluded in a future Number.)*

---

*Prize Questions.* [Inserted by desire of the Secretary to the Academy of Medicine.]

La Société académique de médecine a adopté la série suivante de questions, à la solution desquelles tous les médecins français et étrangers sont appelés à concourir:

I. Donner un précis historique de ce que l'on a écrit de plus positif sur la doctrine du pouls;

Caractériser l'état du pouls propre aux âges, aux constitutions, et aux différens tems des maladies;

Déduire de ces recherches tout ce qui peut éclairer la physique animale, étendre l'art du pronostic, et guider plus sûrement dans la pratique de la médecine.

II. Quelles sont les précautions médicales qu'exigent les grandes opérations de la chirurgie?

Quelle attention doit-on apporter à prescrire les soins de médecine clinique qui peuvent en assurer le succès?

Importe-t-il à l'intérêt de la science et à celui des malades, que le médecin et le chirurgien s'imposent le devoir de s'assister et de s'éclairer mutuellement dans toutes les circonstances essentielles aux opérations?

III. Qu'est-ce que l'on entend par fièvre ataxique?

Y en a-t-il plusieurs espèces?

Ne doit-on pas, selon les différences et les époques de la maladie, varier la méthode de traitement?

IV. Reconnaissant que la fièvre est souvent un effort employé par la nature pour préparer la coction de la maladie, démontrer les dangers du quinquina administré sans discernement.

V. Les matériaux destinés à former le lait sont-ils sécrétés immédiatement des artères par les mamelles; comme le pensent Bichat et M. Chaussier?

On

Ou bien sont-ils apportés aux mamelles par les vaisseaux lymphatiques, comme M. Richerand a cherché à le prouver?

Ou bien encore existe-t-il dans le bas-ventre un organe particulier, jusqu'à présent de nature inconnue, qui fasse subir à ces matériaux un premier degré d'élaboration, avant d'acquérir, dans leur passage par les mamelles, le dernier degré d'assimilation, comme l'a présumé M. Girard, de Lyon, d'après quelques faits particuliers?

VI. Peut-on déduire des conséquences sur les causes du calcul urinaire, de ce que les enfans et les vieillards y sont particulièrement sujets?

VII. Les eaux minérales factices ont-elles les mêmes propriétés que les eaux minérales naturelles, et doivent-elles être indifféremment conseillées; abstraction faite des avantages qui peuvent résulter du voyage à la source, et du séjour dans l'endroit où elle est située?

VIII. La phthisie pulmonaire est-elle contagieuse?

Balancer l'affirmative et la négative de cette question si importante à l'hygiène.

IX. Quelles sont les diverses méthodes de traitement applicables aux diverses périodes de la phthisie pulmonaire?

X. Y a-t-il une ligne de démarcation bien tranchée entre un rhume ordinaire et la phthisie pulmonaire commençante?

Quels sont les signes qui peuvent faire distinguer ces deux affections?

Un diagnostic certain est ici d'une haute importance pour les indications thérapeutiques à remplir.

XI. Etant donné que, dans les maladies en général, la faiblesse, considérée comme symptôme, peut conduire à des erreurs funestes, déterminer d'une manière précise à quels signes on peut reconnaître que la faiblesse est directe ou indirecte.

XII. Constater par des observations, plus exactes que celles qui ont été produites jusqu'ici, quelles sont les propriétés du camphre.

Peut-on concilier ses effets toniques et rehaussant, les propriétés vitales sensiblement diminuées dans la fièvre adynamique, avec ses vertus sédatives et antispasmodiques, dans les fièvres inflammatoires, dans les névroses en général, et, en particulier, dans celles des organes génitaux?

XIII. La dénomination de fièvre adynamique convient-elle spécialement à la fiévre que l'on nommait putride?

Cette dénomination présente-t-elle des avantages? A-t-elle des inconvéniens?

XIV. Ne peut-on pas confondre la rougeole avec d'autres fièvres exanthématiques aiguës?

Etablir comparativement leurs caractères distinctifs.

Cette maladie est-elle sujette à récidive?

XV. La coqueluche est-elle contagieuse ou épidémique?

Dans le premier cas, quel serait son mode de contagion?

Déduire des réponses à ces questions les règles hygiéniques à suivre suivre dans le traitement de cette maladie, relativement surtout à l'usage où l'on est de séquestrer les individus qui en sont atteints.

Quelle est sa durée la plus ordinaire?

Est-elle sujette à récidive?

XVI. Indiquer la différence qui existe entre l'épilepsie, l'hystérie et les convulsions générales ou partielles.

Certaines convulsions des enfans, par exemple, ne peuvent-elles point être considérées comme de véritables accès d'épilepsie?

Quelles sont les meilleures méthodes curatives applicables à cette maladie?

XVII. Quelles sont, d'après l'esprit des langues, les règles et les limites à prescrire relativement à l'innovation des mots, et à l'adoption de nouvelles nomenclatures, dans les diverses branches de la science médicale?

XVIII. L'application topique des dissolutions d'opium convient-elle dans les ophtalmies?

Dans quel cas et à quelle époque faut-il les employer?

XIX. Quelle conséquence fâcheuse dérive de ce préjugé vulgaire, que le *colostrum* n'est pas une nourriture appropriée au nouveau-né, et qu'on doit remédier à ses effets nuisibles, en soumettant l'enfant, aussitôt après la naissance, à l'usage des purgatifs?

XX. Quel est pour l'emploi médical le véhicule le plus convenable du muriate sur-oxidé de mercure, du muriate de baryte et du muriate d'or?

XXI. N'a-t-on pas accordé une confiance trop aveugle à l'usage du lait dans tous les cas d'empoisonnement, surtout dans celui par l'oxide de cuivre ou verdet?

Quel serait, dans ce dernier cas, l'antidote le plus rationnel?

XXII. Exposer les signes caractéristiques de la présence des vers dans le tube intestinal, et ceux qui pourraient faire confondre cette affection avec d'autres analogues.

XXIII. L'opinion de Pouteau et de M. Roussille-Chamseru sur l'existence de luxations musculaires, est-elle fondée?

N'a-t-on pris pour luxation musculaire la saillie de certaines portions de muscles, qui se trouvent en contraction lorsque d'autres sont dans le relâchement?

XXIV. Existe-t-il une fièvre essentielle, particulière aux femmes en couche, ayant son caractère spécifique et ses signes distinctifs, à laquelle la dénomination de *fièvre puerpérale* puisse convenir?

Dans la dernière séance de chaque année académique, la Société, après avoir entendu sa commission des travaux, décernera des récompenses et des encouragemens aux auteurs des meilleures réponses. Elle y joindra un diplôme de correspondant pour ceux qui en feront la demande.

Les mémoires ou observations en réponse aux questions, écrits en français ou en latin, avec les noms des auteurs, seront envoyés francs de port à M. Le Seure, secrétaire de l'Académie de Médecine à l'Oratoire-Saint-Honoré, ou rue des Francs-Bourgeois, n°. 13, au Marais.

La

La Société rappelle que, tout en désirant une correspondance active, elle ne détermine aucune époque pour l'envoi des mémoires, parce qu'elle recônnaît la nécessité de laisser à chacun le tems de se livrer aux recherches et aux expériences qui peuvent jeter du jour sur les questions qu'il se propose de résoudre. Elle permet à chaque auteur de modifier son sujet, d'indiquer de nouveaux points de vue sur des sujets analogues ou différens, de diviser les questions ou d'en réunir plusieurs, suivant qu'il jugera convenable de traiter d'un objet simple ou complexe, et de séparer ou de rapprocher les analogies. Ce ne sont pas des monographies qu'elle demande, mais des réponses exactes et aussi précises que la matière pourra le comporter.

---

The French, with their usual verbiage, have admitted a long paper on Belladonna in Hooping-Cough, as if all the various narcotics had not been already tried in that disease, the pathology of which is now well understood. During the inflammatory period, which continues about a fortnight, the remedies must be directed to that state of the constitution. Afterwards, in this as in all other complaints, we must be governed by symptoms; but, in the chronic state, no remedy has ever been found equal to change of atmosphere,—whether to a purer or not, is often indifferent. But to propose remedies for a disease by its name, is ill suited to the present state of medical science in Great Britain.

---

*Rabies.*—The application of the most powerful caustics, so long recommended in the bite of a mad dog, has been found entirely inefficacious in France; and it is asserted that nothing is to be depended on as certain save the actual cautery, or complete excision of the part by burning with an iron *at a white heat.*

---

*Decoction of white Briony-root, poisonous.*—A French physician was called in to attend a woman in child-bed, whose child was dead. The village surgeon had ordered the mother, to prevent a secretion of milk, to take a decoction of one ounce of white briony root (*Brionia alba,* Lin.), and a *liniment* of a concentrated decoction of the same. On the arrival of the physician, the patient was no more; but, on examining the contents of the evacuation produced by the clyster, he found the internal membrane of the rectum, which had been destroyed by the caustic poison of the decoction of briony. Delicacy, and the reputation of the village surgeon, prevented him from opening the body, in which, he says, he doubts not he should have discovered in the stomach the fatal effects of the medicament.

The root of this plant is too often administered as a strong purgative, but Dr. Orfila has shewn that it killed dogs, by inflammation of the stomach and the lower intestines.

Briony (*bryonia diöica*) is a creeping plant, generally found in hedges. It is constantly diöical, that is, the flowers of the two sexes are placed on different plants. Linnæus has improperly

 confounded confounded it with that growing in the north, which he calls *bryonia alba.* The poisonous principle contained in the root is soluble in water, so that, after frequent washings, the pulp itself may be taken as food.

As to the pellicle taken by the author for the internal membrane of the rectum, it undoubtedly was one of those membraniform concretions produced by the inflammation of the intestinal mucus, and analogous to that formed in the croup [coagulated lymph].

---

*Observations on some Causes of Deafness, with the Means of removing them;* by Professor AUTHENRIETH, of Tubingen.

The closing of the Eustachian tube, which at last causes loss of hearing, so difficultly removed by syringing the tube, and so imperfectly remedied by piercing the tympanum, almost always arises from a catarrhal cause. Twice I have seen this closure occur so frequently at the same time, that one would have been almost justifiable in calling it epidemic. After moderating the determination of blood to the head, and the catarrhal affection, by the usual remedies, the best means of obviating the permanent closing of the tube, even after the hearing has been lost for some weeks, and the forcing of air into the tube by expiring strongly with the mouth and nostrils shut, has been tried in vain, is to excite and maintain for several days, or even weeks, an artificial inflammation of the pharynx, attended with a great discharge of mucus. It acts as an evacuant remedy upon the neighbouring mucous membrane, still turgid with the stagnant fluids produced by the catarrhal inflammation.

Two or three drachms of mezereon bark, boiled down with two ounces of honey and enough of water, to ten ounces of decoction, to which as much caustic ammonia is added as to give the mixture a biting taste upon the tongue, which requires generally from half a drachm to a drachm, is to be used as a gargle every two hours, so as to excite slight inflammation very slowly. If, therefore, upon the first trial, it should seem to act too quickly, it must be diluted with more water, and employed less frequently. In old people, still greater caution is requisite, as in them the inflammation excited is more obstinate, and is apt to be unexpectedly aggravated.

The hearing is frequently not restored until after the inflammation has again ceased.

In the bodies of almost all old people, there is found in the innermost part of the meatus auditorius externus, a firmly attached lump of indurated ear-wax, which, in old age, acquires a disposition to crystallize partly in an earthy form. The ear-picker only scratches off its outer surface. This lump is the cause of the dulness of hearing which occurs slowly in healthy old people. We have as yet no very convenient method of removing it.

The tympanum of the ear is frequently destroyed in consequence of the suppuration of the external meatus auditorius; and of the small bones of the ear, the stirrup only is left, which continues to shut the fenestra ovalis. But the entrance of the cold external air into the cavity of the tympanum, stimulates the membrane lining it. Moist air swells it hygroscopically. Then undulations, which would have excited vibrations in the internal parts of the ear, even without the tympanum, or small bones, are suffocated in it, and such patients become at that time perfectly deaf. The wearing of cotton within the ear, during such weather, preserves from pain, but, on the other hand, renders the feebly observed sounds still more obscure. At these times, it is of use to wear in the meatus an artificial tympanum, consisting of a short tube of lead pressed into an elliptic form, upon the inner end of which the membrane of the swimming bladder of a small fish had been previously stretched when wet, and varnished over after it had become dry.—*Edinb. Med. and Surg. Journal.*

---

We have had the happiness to hear that our late worthy colleague, Dr. S. Fothergill, has very much recovered since his arrival in Jamaica. He has left Kingstone for a distant part of the island, where his professional services may be more required.

---

Mr. Pettigrew is preparing for publication, Memoirs of the Life and Writings of the late John Coakley Lettsom, M. and LL.D. F.R.S. F.A.S. F.L.S. &c. &c. &c. with a Selection from his Correspondence with the principal Literati of this and foreign Countries.—It is intended to comprise this work in three octavo volumes, and to publish them by subscription. The two first volumes will consist of a Memoir of Dr. Lettsom, drawn from original and authentic sources; and of a selection from his very extensive correspondence, principally with the following distinguished characters: viz. Baron Haller, Linnæus, Lord Lansdown, Dr. Cullen, and nearly one hundred others in various parts of the globe.

---

Mr. John Mason Good has in the press, a work to be entitled a Physiological System of Nosology, with a simplified and corrected Nomenclature, &c. The whole will form one volume octavo.

---

In a few weeks will be published, a Compendium of Medical Surgery, translated and abridged from Depelch Elementaire des Maladies. The work will include the modern English practice in this branch of medical science, and the whole so condensed as to form an interesting and useful volume.

---

In a few days will be published, the second part of Transactions of the Medical Society, containing Dr. Adams's paper on Epilepsy, Dr. Blegborough's on Croup, &c.

### METEOROLOGICAL REGISTER.

*From September the 25th, to October the 25th,* 1816.

Kept by C. BLUNT, Philosophical Instrument Maker, No. 38, Tavistock-Street, Covent-Garden.

| Moon. | Day. | Wind. | Barometrical Pressure. Max. | Min. | Mean. | Temperature. Max. | Min. | Mean. | |
|---|---|---|---|---|---|---|---|---|---|
| | 26 | S | 29·80 | 29·60 | 29·70 | 66 | 44 | 55· | Fair |
| | 27 | S | 30·10 | 29·62 | 29·86 | 67 | 44 | 55·5 | Fair |
| ☽ | 28 | SW | 30·30 | 29·63 | 29·96 | 66 | 45 | 55·5 | Fair |
| | 29 | W | 30· | 29·50 | 29·75 | 65 | 43 | 54· | Fair |
| | 30 | W | 30·10 | 29·50 | 29·80 | 64 | 42 | 53· | Rain |
| | 1 | SW | 30·20 | 30· | 30·10 | 62 | 43 | 52·5 | Rain |
| | 2 | SW | 29·90 | 29·80 | 29·85 | 61 | 41 | 51· | Fair |
| | 3 | W | 30·20 | 30·10 | 30·15 | 63 | 42 | 52·5 | Fair |
| | 4 | NW | 30· | 30· | 30· | 60 | 40 | 50· | Rain |
| | 5 | NW | 29·98 | 29·96 | 29·97 | 59 | 38 | 48·5 | Rain |
| ○ | 6 | NNW | 29·99 | 29·96 | 29·93 | 61 | 40 | 50·5 | Rain |
| | 7 | N | 29·93 | 29·90 | 29·92 | 59 | 39 | 49· | Rain |
| | 8 | NW | 30· | 29·91 | 29·95 | 59 | 39 | 49· | Fair |
| | 9 | N | 30·04 | 30· | 30·02 | 58 | 39 | 48·5 | Fair |
| | 10 | NE | 30·03 | 30· | 30·02 | 59 | 45 | 52· | Fair |
| | 11 | E | 30·13 | 30· | 30·06 | 59 | 44 | 51·5 | Fair |
| | 12 | SE | 30·24 | 30·10 | 30·17 | 61 | 42 | 51·5 | Fair |
| | 13 | SE | 30·17 | 30· | 30·08 | 63 | 41 | 52· | Fair |
| ☾ | 14 | S | 30·19 | 30· | 30·09 | 62 | 40 | 51· | Fair |
| | 15 | S | 30·18 | 29·90 | 30·04 | 64 | 43 | 53·5 | Fair |
| | 16 | S | 30·05 | 29·80 | 29·92 | 63 | 41 | 52· | Fair |
| | 17 | S | 30·01 | 30· | 30· | 62 | 40 | 51· | Fair |
| | 18 | SE | 29·99 | 29·94 | 29·96 | 59 | 38 | 48·5 | Rain |
| | 19 | SE | 29·95 | 29·90 | 29.92 | 60 | 40 | 50· | Rain |
| ● | 20 | E | 29·93 | 29·80 | 29·86 | 58 | 36 | 47· | Fair |
| | 21 | E | 29·85 | 29·80 | 29·83 | 57 | 35 | 46· | Fair |
| | 22 | E | 29·73 | 29·70 | 29·71 | 55 | 34 | 44·5 | Rain |
| | 23 | E | 29·74 | 29·71 | 29·72 | 56 | 36 | 46· | Fair |
| | 24 | E | 29·75 | 29·72 | 29·73 | 56 | 38 | 47· | Rain |
| | 25 | E | 29·76 | 29·74 | 29·75 | 58 | 38 | 48· | Rain |

RESULTS.

Mean barometrical pressure of the month 29·75
Maximum 30·30, wind at SW
Minimum 29·50, ——— W

Mean temperature of the month 51 deg.

Maximum 67, wind at S

Minimum 34, ——— E

*Scale exhibiting the prevailing Winds during the Month.*

| N | NE | E | SE | S | SW | W | NW |
|---|---|---|---|---|---|---|---|
| 2 | 1 | 7 | 4 | 6 | 3 | 0 | 4 |

| | Mean barometrical pressure. | Mean temperature. |
|---|---|---|
| From the first quarter on the 28th Sept. to the full moon on the 6th Oct. | 30·10 | 51·8 |
| ——— the full moon on the 6th, to the last quarter on the 14th | 30·24 | 50·6 |
| ——— the last quarter on the 14th, to the new moon on the 20th | 29·90 | 51·42 |
| ——— the new moon on the 20th, to the 25th, the close of the period | 29·77 | 46·3 |

REPORT

### REPORT OF DISEASES.

The town is so remarkably healthy, that, as Sydenham used to remark, the contagions bear their full sway. Small-pox extends as far as it is permitted, which is much too far: it is, however, much milder than during the preceding month. Scarlatina is very general, and very severe; but not often fatal. The chief chronic diseases are the sequelæ of ill-treated or neglected visceral inflammations during the spring and summer. The autumnal epidemics rarely appear. Dysentery is unknown in London; and diarrhœa so mild as scarcely to require any remedy. Inflammation sometimes appears, principally about the head, but with none of its former severity.

