# Corrigenda

**Published:** 1816-12

**Authors:** 


					p. 429,1. 5 from the bottom, for j alap read soup*

				

